# The Efficacy of Different Chemotherapy Regimens for Advanced Biliary Tract Cancer: A Systematic Review and Network Meta-Analysis

**DOI:** 10.3389/fonc.2019.00441

**Published:** 2019-05-29

**Authors:** Yan Li, Yaoyao Zhou, Yonglan Hong, Meizhi He, Shuyi Wei, Chen Yang, Dayong Zheng, Feiye Liu

**Affiliations:** ^1^Integrated Hospital of Traditional Chinese Medicine, Southern Medical University, Guangzhou, China; ^2^The Second School of Clinical Medicine, Southern Medical University, Guangzhou, China; ^3^The First School of Clinical Medicine, Southern Medical University, Guangzhou, China; ^4^The First Affiliated Hospital of Guangzhou University of Chinese Medicine, Guangzhou University of Chinese Medicine, Guangzhou, China

**Keywords:** biliary tract cancer, chemotherapy, Folfox-4, network meta-analysis, efficacy

## Abstract

**Background:** Although gemcitabine plus cisplatin (GP) is considered as standard chemotherapy for patients with advanced biliary tract cancer (BTC), the optimal regimen remains unknown.

**Methods:** Using Network meta-analysis (NMA), a systematic review was conducted to find the most effective chemotherapy regimen for advanced BTC. We searched PubMed, Web of Science, Embase, Scopus and the Cochrane Library for articles published before October 6, 2018. Articles about chemotherapeutic comparisons were included. Hazard ratios (HRs) for overall survival (OS) and progression free survival (PFS) were estimated while odd ratios (ORs) was assessed for objective response rate (ORR).

**Results:** The NMA included 25 studies and 3,312 individuals. Among all the regimens, Folfox-4 regimen obtained a superior difference in OS (BSC vs. Folfox-4, HR 3.4, 95% CI 1.7-6.7). XP was slightly better than GP in OS and GS approximately obtained the same efficacy to GP (HR for XP vs. GP 0.74, 95% CI 0.51-1.1; HR for GS vs. GP 1.1, 95% CI 0.71-1.5). Most of the targeted therapies included in this study tend to achieve better results in PFS and ORR but failed to improve OS, in which E-GEMOX achieved the best ORR when compared to BSC (OR 0.03, 95% CI 0.00-0.94).

**Conclusions:** Folfox-4 regimen is likely to be the optimal chemotherapy for patients with advanced BTC and the predominant targeted therapy hasn't achieved significant success currently. XP and GS can be considered as alternatives for advanced BTC.

## Introduction

Biliary tract cancer (BTC) is a heterogeneous group of malignancies with features of biliary tract differentiation arising from distinct anatomical locations of the biliary tree. BTC is generally classified as intrahepatic cholangiocarcinoma, extrahepatic cholangiocarcinoma, gallbladder carcinoma, and ampullary cancer. Biliary tract cancer is a rare tumor in some European countries and the United State, but with a higher incidence in Latin America and Asia (1–4). BTC is highly fatal malignancy with a low detection and a poor prognosis, which means that a surgical resection is difficult to carry out when patients are diagnosed (5, 6). Therefore, palliative chemotherapy becomes a more essential treatment to improve patients' survival and quality of life. Gemcitabine plus cisplatin (GP) chemotherapy is considered to be the standard first-line chemotherapeutic regimen for advanced BTC after ABC-02 trials (7) and BT22 trials were conducted (8). The two trials both demonstrated that GP was associated with a significant survival advantage without increasing adverse events. Fluorouracil based chemotherapy including S-1 and 5-FU is also confirmed to be an effective therapy to treat advanced BTC (9, 10) with less adverse events than gemcitabine (GEM) based (11). There are currently several trials investigating the role of molecularly targeted drugs plus gemcitabine and oxaliplatin (GEMOX) in BTCs (12–15). Nevertheless, the efficacy of targeted therapy remained controversial. Two traditional meta-analysis articles had revealed that the targeted therapy did improve the objective response rate (ORR) apparently and drugs targeting EGFR obtained a superior progression free survival (PFS) instead of VEGF, but all failed to prolong patients' survival (16, 17). No significant difference was observed in the incidences of the grade 3/4 adverse events in these two meta-analyses, but patients who received GEMOX plus EGFR-targeted chemotherapy showed higher risks of diarrhea, neutropenia, transaminase increase and a skin rash than those who were treated with GEMOX chemotherapy in the meta-analysis of four randomized control trials (RCTs). A NMA suggested that targeted agents added to GP or GEMOX achieved a superior survival outcome though associated with higher risk of adverse effects (11). A randomized phase II study conducted by Schinizari et al. in 2017 suggested that 5-FU/LV plus oxaliplatin (Folfox-4) could be an option as first-line treatment with the median overall survival (OS) of 13.0 months (9), even though the regimen of 5-FU and folinic acid (FUFA) were ever considered to be less effective (18). However, due to limitations of traditional meta-analysis and the lack of direct comparison trials, the most effective regimen for BTC remains unknown. NMA is a better way to identify that regimen with the advantage of combining direct and indirect evidence, allowing no head-to-head comparisons from different trials (19).

Therefore, we used NMA of randomized controlled trials and retrospective studies in this study to find the optimal regimen for advanced BTC.

## Methods

This NMA was conducted and reported on the recommendations of the preferred reporting items for systematic reviews and meta-analyses (PRISMA) guidelines (20).

### Search Strategy

A literature search was conducted for all articles published before October 6, 2018 using PubMed, Web of Science, Embase, Scopus and the Cochrane Library. The following search terms were used separately or in combination: (biliary tract neoplasm OR biliary Tract neoplasm OR neoplasm, biliary tract OR neoplasms, biliary tract OR biliary tract cancer OR biliary tract cancers OR cancers, biliary tract OR cancer of biliary tract OR cholangiocarcinoma OR gallbladder neoplasms) AND (chemotherapy OR fluorouracil OR gemcitabine OR CRT OR panitumumab OR oxaliplatin OR cisplatin). Additionally, we manually searched bibliographies and added related references. Two investigators (YY Zhou and YL Hong) independently reviewed the titles and abstracts. Any disagreements were resolved by discussion and consultation.

### Eligible Criteria

The selection criteria and exclusion criteria were predefined. Articles were included if they met the following inclusion criteria: (1) randomized controlled trials or retrospective studies; (2) patients with advanced biliary tract cancer who received chemotherapy; (3) reported outcome values (PFS, OS, and/or ORR). The exclusion criteria were as followed: (1) letter, commentaries, case reports, reviews, non-human studies and articles that did not provide raw data; (2) non-English articles; (3) articles that were not randomized or retrospective studies; (4) studies that did not include chemotherapeutic comparison; (5) studies included chemotherapeutic comparison concerning adjuvant therapy or new-adjuvant therapy. If multiple publications from the same study were available, we included the publication with the largest number of cases and the most applicable information.

### Data Extraction

Two investigators (YY Zhou and YL Hong) independently reviewed the full text and extracted the following information: (1) publication data: first author name, publication year, country, and period of recruitment; (2) characteristics of each study population: number of patients, median age, and gender; (3) tumor stage and the ECOG performance status of patients; (4) treatment characteristics: regimen, dosage, cycles and median follow-up period; (5) outcome: PFS, OS, and ORR. When HRs was not reported in the articles, digitizing software (Digitizeit) was used to extract the HRs from the survival curve with a high degree of accuracy following established methods (21).

### Statistical Analysis

Data was entered and analyzed using Review Manager Version 5.3 (Revman the Cochrane Collaboration; Oxford, England). Time-related endpoints (PFS and OS) were reported as hazard ratios (HRs) with 95% confidence intervals (95% CI) which were used to calculate the efficacy of different chemotherapy for BTC. HRs was obtained directly from the articles or used Kaplan-Meier survival curves to estimate, while ORR and odd ratios (ORs) were used to assess the pharmacological effects. Pairwise comparisons were made by combining studies that compared the same intervention. Statistical heterogeneity from the eligible studies was assessed with the Chi^2^ test and the I^2^ statistic. The *P*-value > 0.10 for the Chi^2^ test or I^2^ >50% implied heterogeneity, thus a random effects method was required, or else a fixed effects method was needed. In this study, heterogeneity was found in the direct comparison of two articles and a random effects method was used.

A Bayesian framework and Markov chain Monte Carlo methods were used for a random-effects model in indirect comparison meta-analysis (22). The NMA, consisting of direct and indirect comparison, can be considered as an extension of the traditional meta-analysis. By obtaining the comparison of A to B and B to C, NMA makes it possible to estimate the benefit of A over C that can't be compared directly. A network plot was constructed to present all the relationships of the included regimens. NMA is based on three main assumptions. First, all the studies enrolled are homogeneous. In our study, we found heterogeneity in one direct comparison concerning two studies and we dealt with it by using a random effects model. Then all trials in each study are similar in both clinic and methodology ignoring the differences produced by age, gender, region and the criteria of final end point. Last, the results of direct and indirect evidence are consistent. The size of the node represents the proportion of the patients receive the treatment and the width of the lines is proportional to the number of the trials comparing the connected treatments. The NMA also allows ranking of the enrolled treatments. To obtain the ranking probability of the different regimens, the surface under the cumulative ranking curve (SUCRA) was calculated (23). The SUCRA index ranges between 0 (or 0%) and 1 (or 100%), where the treatments with higher SUCRA values are considered to have better efficacy.

## Results

### Description of Eligible Trials

**Figure 1:**
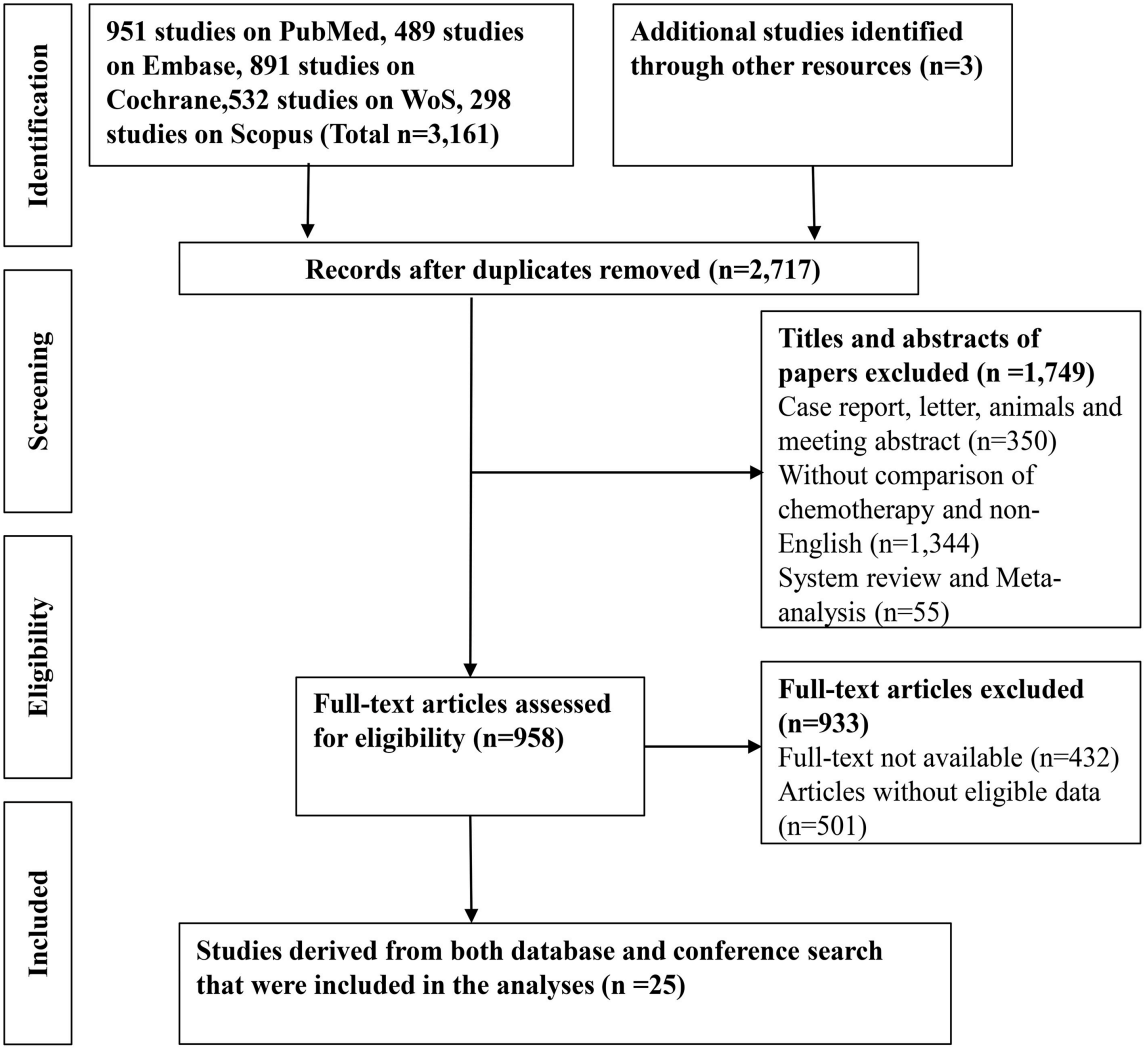
A flow-chart of the literature search strategy and included studies in this network meta-analysis.

A total of 3161 records were identified through searching PubMed, Embase, WoS, Scopus and the Cochrane Library. Three additional articles were added from references. 487 records were removed due to duplications. 1749 articles were excluded after reviewing title and abstract due to: (1) articles were reviews, letters, meetings, and case reports; (2) the research object was not human; (3) articles did not concern chemotherapeutic comparison. Full-texts of the 958 articles were assessed and 933 articles were excluded as they were not available, contained no eligible data, contained duplication of data, and/or were not suitable for analysis. Therefore, 25 RCTs and retrospective articles were included in this NMA (Figure 1). 3,312 patients in all were enrolled in the 25 studies of which 13 were RCTs and eight were retrospective studies. There were six studies conducted in Korea (13, 24–28), five in Japan (8, 29–32), four in Italy (9, 15, 33, 34), two in China (35, 36), two in the UK (7, 37), two in France (38, 39), one in India (40), two in Germany (14, 41), and one in France and Germany (12). The recruitment period ranged from 1998 to 2015 while the patients number ranged from 34 to 410 and the median age varied from 47 to 75 (Table 1). Gemcitabine was generally used at a dose of 1,000 mg/m^2^ with the combination of cisplatin at a dose of 25 mg/m^2^, which was considered as a standard GP regimen. The dose of oxaliplatin was commonly 100 mg/m^2^ while the other drugs differed. The regimens' cycles differed and Most of the studies got outcomes of OS and PFS. However, clinical stage was not explained clearly in most of the including studies (Table 2).

**Table 1:** Characteristic of the included studies.

**References**	**Country**	**Type of study**	**Recruitment period**	**Case**	**Median age, range(years)**	**Gender (male/female)**	**Median follow up, range(months)**
Chen et al. (35)	China	RCT	2010.12–2012.03	122	C-GEMOX: 61 (32–78) GEMOX: 59 (32–80)	58/64	10.1 (0.9–24.4)
Fiteni et al. (38)	France	Retro	1998–2010	64	NA	38/26	NA
Kang et al. (28)	Korea	RCT	2008.03–2009.03	96	GP: 59 (32–77) SP: 60 (36–77)	62/34	14.2 (12.7–15.5)
Kim et al. (27)	Korea	Retro	2001.03–2012.03	92	XPRT: 56 (32–75) XP: 58 (26–78)	72/20	5.3
Park et al. (24)	Korea	Retro	2011.01–2012.04	134	61.0 (36–77)	73/61	26.2 (24.2–28.2)
Lee et al. (13)	Korea	RCT	2009.02.16–2010.08.1	268	GEMOX: 61 (55–68) E-GEMOX: 59 (54–66)	170/98	15
Lee et al. (25)	Korea	Retro	2009.10–2012.07	93	GP: 62 (45–81) XP: 65 (39–80)	61/32	NA
Lenoe et al. (15)	Italy	RCT	2010.06–2013.09	89	Pa-GEMOX: 63.9 (46.7–78.5) GEMOX: 64.2 (36.8–78.5)	32/57	10.1
Li et al. (36)	China	RCT	NA	75	NA	NA	24
Malka et al. (12)	France and Germany	RCT	2007.10.10–2009.12.18	150	C-GEMOX: 61 (35–75) GEMOX: 62 (39–75)	85/65	C-GEMOX: 31.1 GEMOX: 34.9
Moehler et al. (14)	Germany	RCT	NA	97	GSo: 64.0 (44–83) GEM: 64.5 (36–84)	54/43	12
Morizane et al. (32)	Japan	RCT	2009.02–2010.04	101	GS: 66 (39–78) S-1: 62.5 (49–79)	55/46	10.6
Novariono et al. (34)	Italy	Retro	2001–2006	40	Folfox-4: 62(47–75) GEM: 65 (52–75)	17/23	12
Okusaka et al. (8)	Japan	RCT	2006.09–2008.10	83	GP: 65.0 (43–80) GEM: 66.5 (49–78)	39/44	NA
Phelip et al. (39)	France	RCT	2006.07–2010.12	34	FUPR: 69.5 (53–80) GEMOX: 75 (54–81)	15/119	27.9 (19.8–35.9)
Woo et al. (26)	Korea	Retro	2001.11–2012.08	344	GP: 62.0 (35–76) XP: 58.0 (27–82)	206/138	8.9 (0.4–61)
Santoro et al. (33)	Italy	RCT	2008.10–2012.09	173	63.6	92/81	V: 7 (1–38) GV: 8.5 (1–31) GEM: 8 (1–35)
Sasaki et al. (30)	Japan	RCT	2008.11–2010.03	62	GS: 48 (47–83) GEM: 75 (55–86)	36/26	NA
Schinzari et al. (9)	Italy	RCT	NA	48	NA	NA	NA
Sharma et al. (40)	India	RCT	2006.06–2008.10	81	BSC: 51 FUFA: 47 GEMOX: 49	16/65	9 (1–26)
Takahara et al. (31)	Japan	Retro	2006.07–2015.08	212	GS: 68 (24–85) GP: 69 (37–85)	82/130	5.1 (0–34.4)
Valle et al. (7)	UK	RCT	2002.02–2008.10	410	GEM: 63.2 (23.4–98.4) GP: 63.9 (32.8–81.9)	194/216	8.2
Valle et al. (37)	UK	RCT	2011.01.05–2012.09.28	124	GPCe: 68.0 (60.4–73.0) GP: 64.5 (59.7–73.1)	62/62	12.2
Vogel et al. (41)	Germany	RCT	2011.07–2015.12	90	61.5 (18–82)	50/40	NA
Yonemoto et al. (29)	Japan	Retro	2000.04–2003.03	230	NA	133/97	4.57 (0.10–52.57)

*RCT, randomized controlled trial; Retro, retrospective study; NA, not available; GEM, gemcitabine; OX, oxaliplatin; C, cetuximab; E, erlotinib; Pa, panitummumab; GC, gemcitabine+carboplatin; GP, gemcitabine+cisplatin; SP, S-1 +cisplatin; XP, capecitabine +cisplatin; RT, radiotherapy; GSo, gemcitabine+sorafenib; GS, gemcitabine+S-1; Folfox-4, 5-FU+folinic acid+oxaliplatin; FUPR, 5-FU+cisplatin+radiotherapy; FUFA, 5-FU+folinic acid; Ce, cediranib; V, vandetanib; GV, vandetanib+gemcitabine; BSC, best support care*.

**Table 2:** Treatment characteristic of the included studies.

**Study**	**Clinical stages**	**Regimens**	**Dosage**	**No. of cycles**	**ECOG PS**	**Outcomes**
Chen et al. (35)	T1-4, N0-1	C-GEMOX GEMOX	Cetuximab:500 mg/m^2^; gemcitabine: 800 mg/m^2^; oxaliplatin:85 mg/m^2^	NA	0/1	OS; PFS; ORR
Fiteni et al. (38)	NA	GEMOX GC	Gemcitabine: 1000 mg/m^2^; oxaliplatin: 100 mg/m^2^; carboplatin: according to an area-under-the-curve	7	0/1/2/3	OS; PFS; ORR
Kang et al. (28)	NA	GP SP	Gemcitabine: 1000 mg/m^2^; cisplatin: 60 mg/m^2^; S-1: 40 mg/m^2^	6	0/1/2	OS; PFS; ORR
Kim et al. (27)	IVa, IVb	XPRT XP	Capecitabine: 1000 mg/m^2^; cisplatin: 30 mg/m^2^; radiotherapy: 25-60 Gy	XPRT: 6 XP: 4	0/1/2	ORR
Park et al. (24)	NA	XP GP	Gemcitabine: 1000 mg/m^2^; cisplatin: 25/60 mg/m^2^; capecitabine: 1000 mg/m^2^;	XP: 6 GP: 4	0/1/2	OS; PFS; ORR
Lee et al. (13)	NA	GEMOX E-GEMOX	Gemcitabine: 1000 mg/m^2^; oxaliplatin: 100 mg/m^2^; erlotinib: 100 mg/day	GEMOX: 6 E-GEMOX: 7	0/1/2	OS; PFS; ORR
Lee et al. (25)	NA	GP XP	Gemcitabine: 1000 mg/m^2^; cisplatin: 75/60 mg/m^2^; capecitabine: 1250 mg/m^2^;	GP: 4 XP: 3	NA	ORR
Lenoe et al. (15)	NA	Pa-GEMOX GEMOX	Gemcitabine: 1000 mg/m^2^; oxaliplatin:100 mg/m^2^; panitumumab: 6 mg/kg	12	0/1/2	OS; PFS; ORR
Li et al. (36)	NA	GS GEM S-1	Gemcitabine: 1000 mg/m^2^; S-1: 80/100/120 mg/d	NA	NA	OS; PFS; ORR
Malka et al. (12)	NA	C-GEMOX GEMOX	Cetuximab: 500 mg/m^2^; gemcitabine: 1000 mg/m^2^; oxaliplatin: 100 mg/m^2^	10	0/1	OS; PFS; ORR
Moehler et al. (14)	NA	GSo GEM	Gemcitabine: 1000 mg/m^2^; soragenib: 400 mg/day	4	0/1/2	OS; PFS
Morizane et al. (32)	II / III /IV /recurrent	GS S-1	Gemcitabine: 1000 mg/m^2^; S-1(combination): 60 mg/m^2^; S-1(monotherapy): 80 mg/m^2^	GS: 10 S-1: 3	0/1	OS; PFS
Novariono et al. (34)	NA	Folfox-4 GEM	Oxaliplatin: 85 mg/m^2^; folinic acid: 200 mg/m^2^; 5-FU: 400/600 mg/m^2^; gemcitabine: 1250 mg/m^2^	Folfox-4: 6 GEM: 3	0/1/2	OS; PFS; ORR
Okusaka et al. (8)	II/IIIA-C/IV/recurrent	GP GEM	Gemcitabine: 1000 mg/m^2^; cisplatin: 25 mg/m^2^	GP: 4 GEM: 3	0/1	OS; PFS; ORR
Phelip et al. (39)	NA	FUPR GEMOX	5-FU: 300 mg/m^2^; cisplatin: 80 mg/m^2^; radiotherapy: 50 Gy; gemcitabine: 1000 mg/m^2^; oxaliplatin: 100 mg/m^2^	NA	0/1/2	OS; PFS
Woo et al. (26)	NA	XP GP	Gemcitabine: 1000 mg/m^2^; cisplatin: 25/30 mg/m^2^; capecitabine: 1000 mg/m^2^;	3	NA	OS; ORR
Santoro et al. (33)	NA	V GV GEM	Vandetanib: 300 mg/m^2^; vandetanib(combination): 100 mg/m^2^; gemcitabine: 1000 mg/m^2^	6	0/1/2	PFS
Sasaki et al. (30)	NA	GS GEM	Gemcitabine: 1000 mg/m^2^; S-1: 40 mg/m^2^	NA	0/1/2	OS; ORR
Schinzari et al. (9)	NA	Folfox-4 FUFA	NA	NA	NA	OS; PFS
Sharma et al. (40)	NA	BSC FUFA GEMOX	5-FU: 425 mg/m^2^; folinic acid: 20 mg/m^2^; gemcitabine: 900 mg/m^2^; oxaliplatin: 80 mg/m^2^	6	0/1/2	OS; PFS; ORR
Takahara et al. (31)	NA	GS GP	Gemcitabine: 1000 mg/m^2^; S-1: 80 mg/m^2^; cisplatin: 25 mg/m^2^	GS: 4 GP: 5	0/1/2	OS; PFS; ORR
Valle et al. (7)	NA	GEM GP	Gemcitabine: 1000 mg/m^2^; cisplatin: 25 mg/m^2^;	6	0/1/2	OS; PFS
Valle et al. (37)	NA	GPCe GP	Gemcitabine: 1000 mg/m^2^; cisplatin: 25 mg/m^2^; cediranib: 20 mg/day	8	0/1	OS; PFS
Vogel et al. (41)	M0, M1, MX	GPPa GP	Gemcitabine: 1000 mg/m^2^; cisplatin: 25 mg/m^2^; panitumumab: 9 mg/kg	GPPa: 8 GP: 6	0/1/2	OS; PFS
Yonemoto et al. (29)	NA	S-1 GEM FAM BSC	NA	NA	0/1/2/3/4	OS

*ECOG PS, Eastern Cooperative Oncology Group Performance Status; NA, not available; GEM, gemcitabine; OX, oxaliplatin; C, cetuximab; E, erlotinib; Pa, panitummumab; GC, gemcitabine+carboplatin; GP, gemcitabine+cisplatin; SP, S-1 +cisplatin; XP, capecitabine +cisplatin; RT, radiotherapy; GSo, gemcitabine+sorafenib; GS, gemcitabine+S-1; Folfox-4, 5-FU+folinic acid+oxaliplatin; FUPR, 5-FU+cisplatin+radiotherapy; FUFA, 5-FU+folinic acid; Ce, cediranib; V, vandetanib; GV, vandetanib+gemcitabine; FAM, 5-FU+doxorubicin+mitomycin C; BSC, best support care; OS, overall survival; PFS, progression free survival; ORR, objective response rate*.

### Pairwise Meta-Analysis for Efficacy and Response

**Figure 2:**
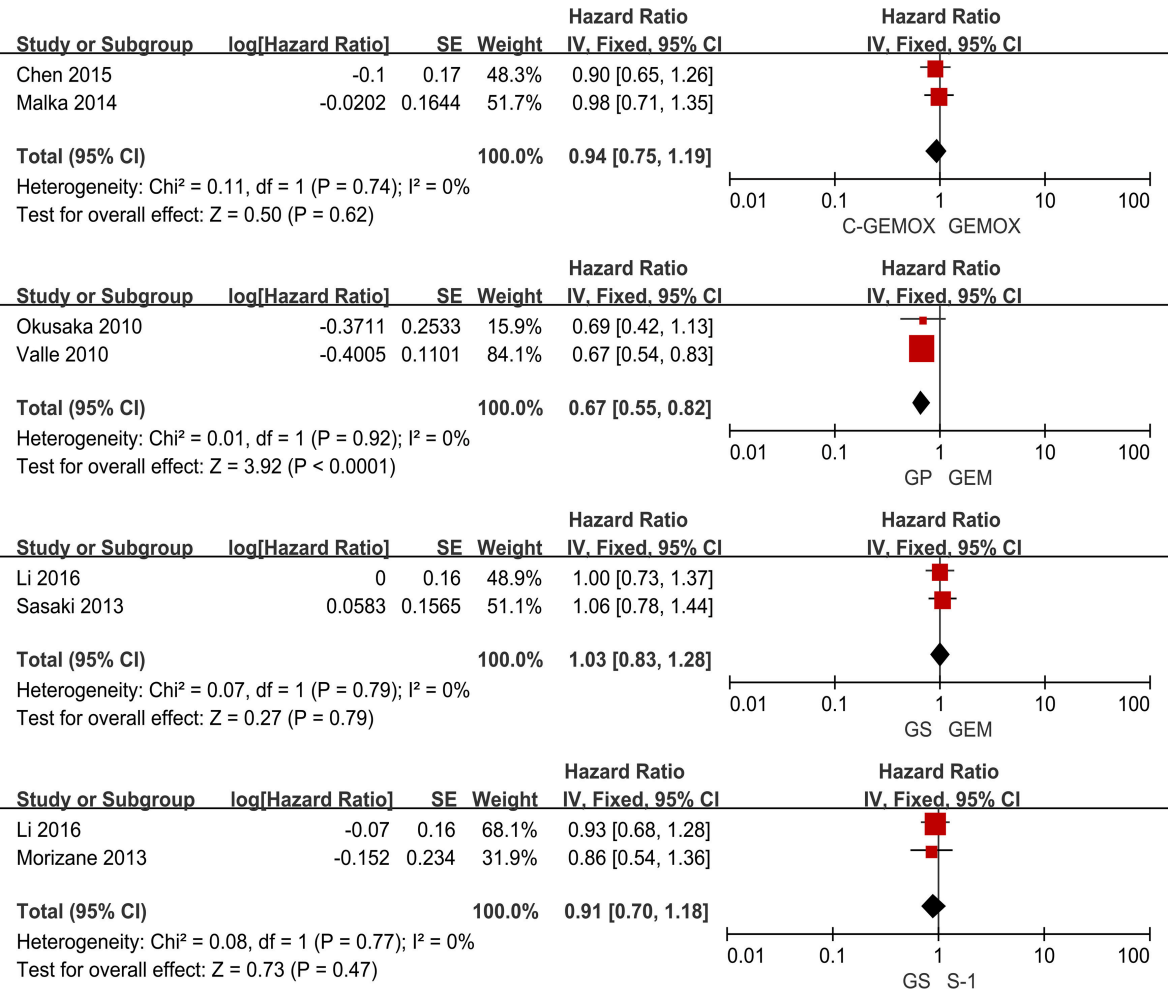
Forest plots of treatments effects and response for overall survival.

**Figure 3:**
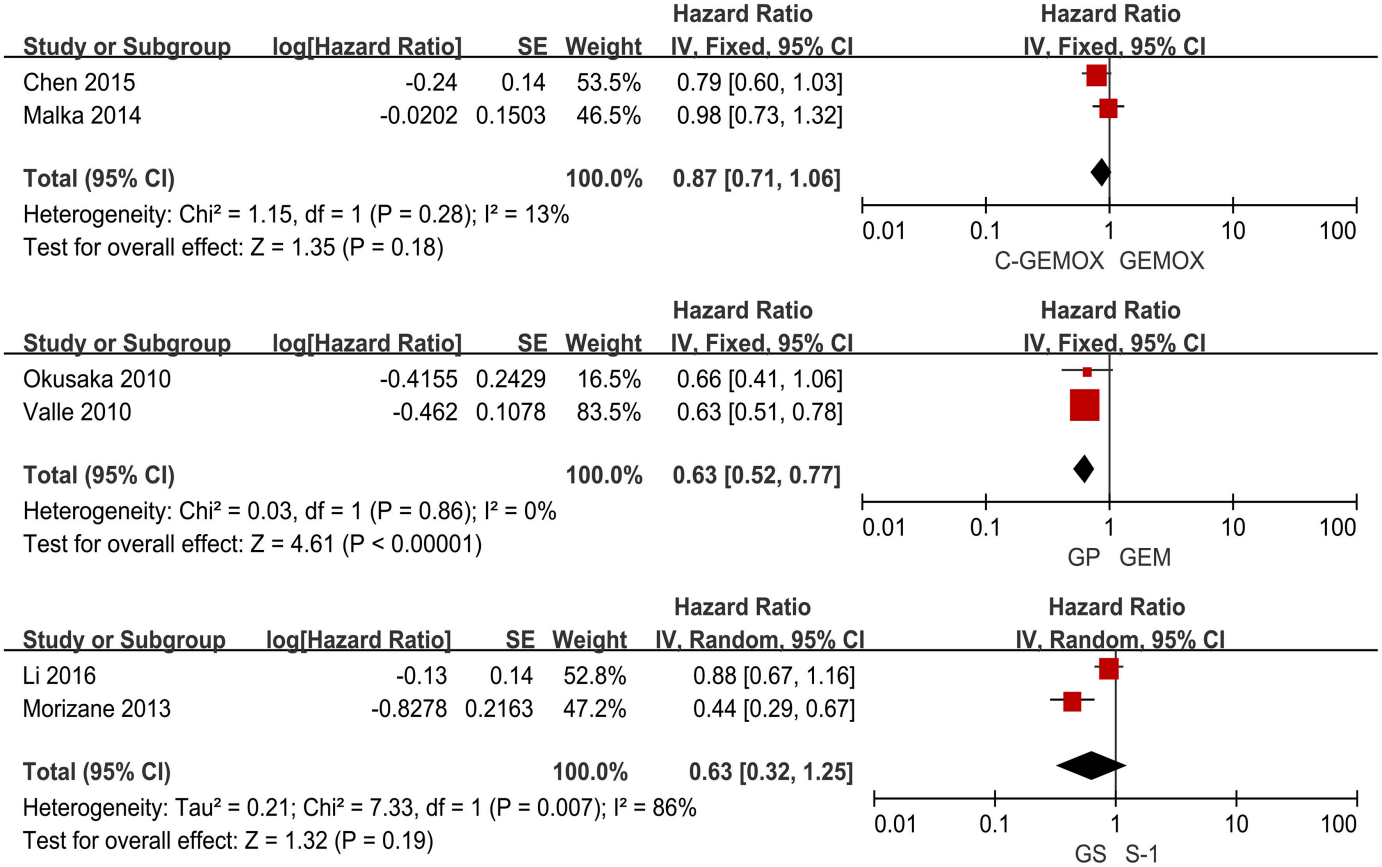
Forest plots of treatments effects and response for progression free survival.

**Figure 4:**
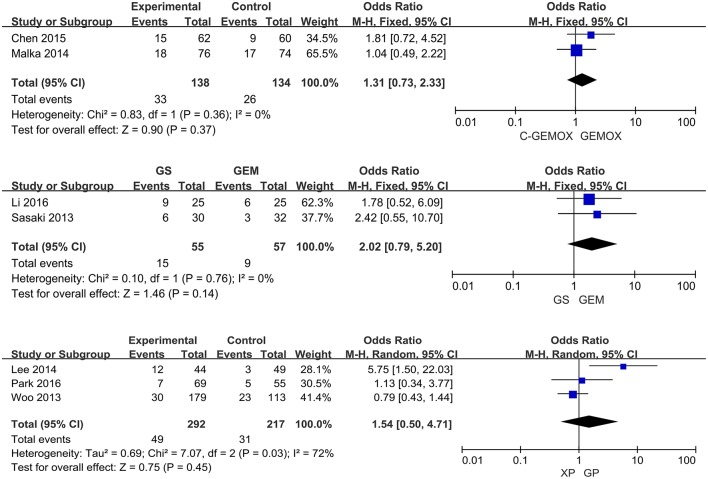
Forest plots of treatments effects and response for objective response rate.

A total of seven studies including 1,003 patients were available for the meta-analysis for OS. As shown in Figure 2, the pooled analysis of OS indicated that GP was significantly associated with better OS than GEM (HR 0.67, 95% CI 0.55-0.82, *p* < 0.0001) while no significant difference was observed when cetuximab plus GEMOX (C-GEMOX) was compared to GEMOX, gemcitabine plus S-1 (GS) to GEM and GS to S-1. The pooled analysis of PFS was based on six studies enrolling 941 patients. Figure 3 showed that significant difference could be observed when GP compared to GEM (HR 0.63, 95% CI 0.52-0.77, *p* < 0.00001). Seven studies were included for pooled analysis of ORR with 980 patients. From Figure 4, no significant heterogeneity was observed among the included studies for ORR.

### Network Meta-Analysis for Efficacy and Response

**Figure 5:**
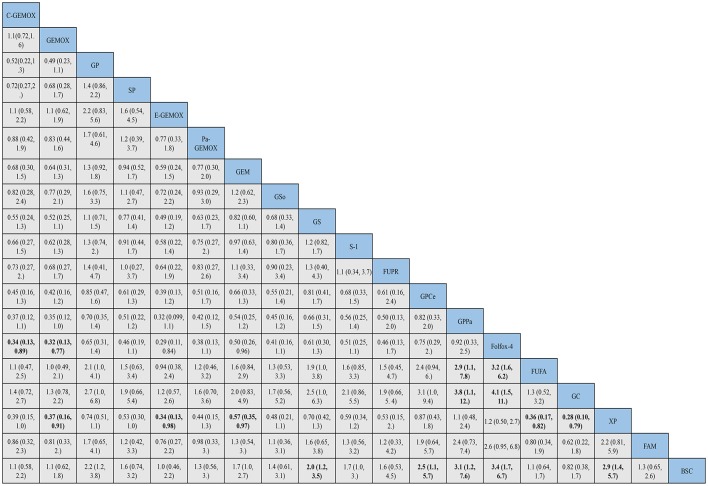
Bayesian framework network meta-analysis. Hazards ratio and 95% confidence intervals for overall survival.

**Figure 6:**
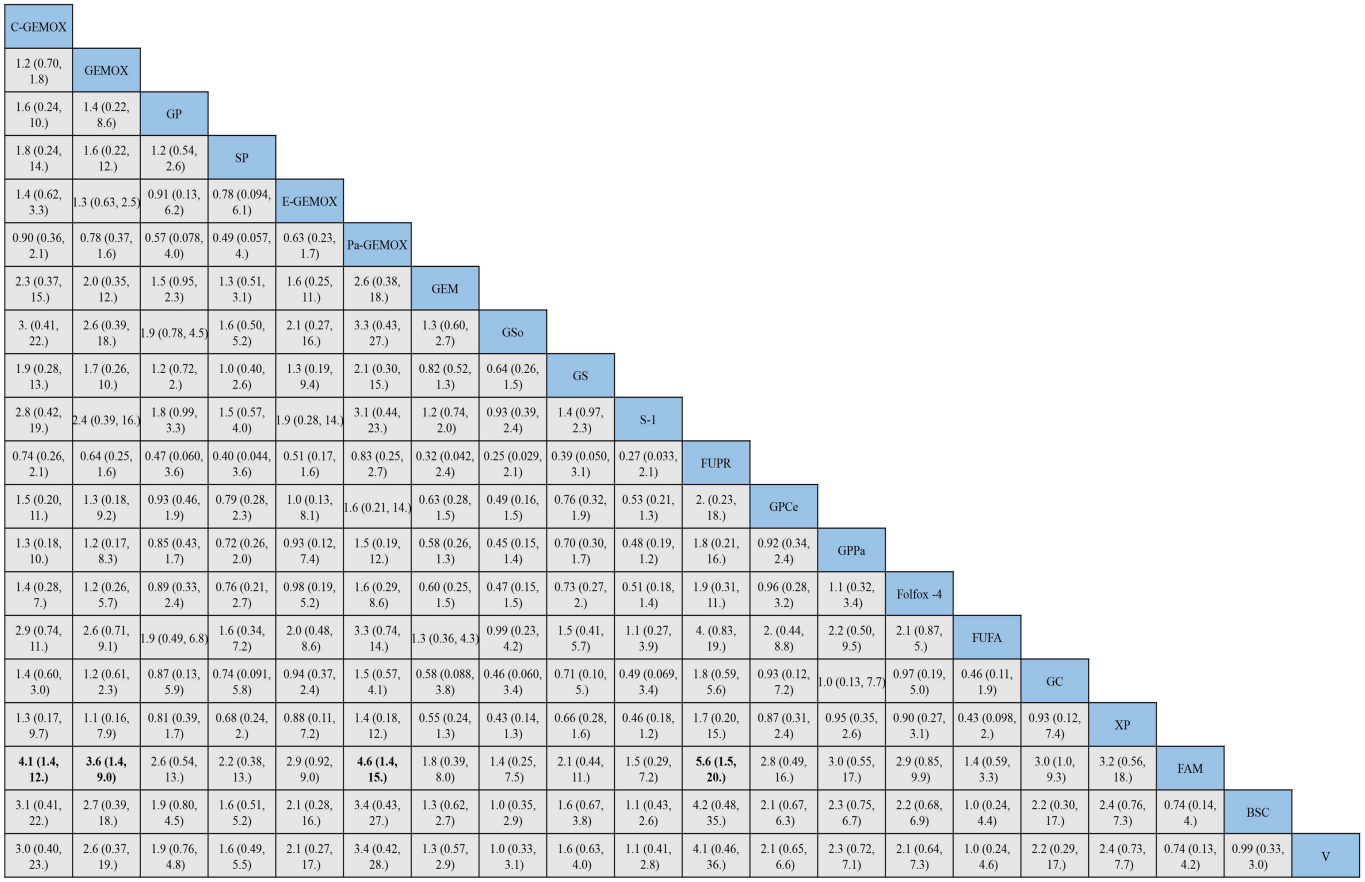
Bayesian framework network meta-analysis. Hazards ratio and 95% confidence intervals for progression free survival.

**Figure 7:**
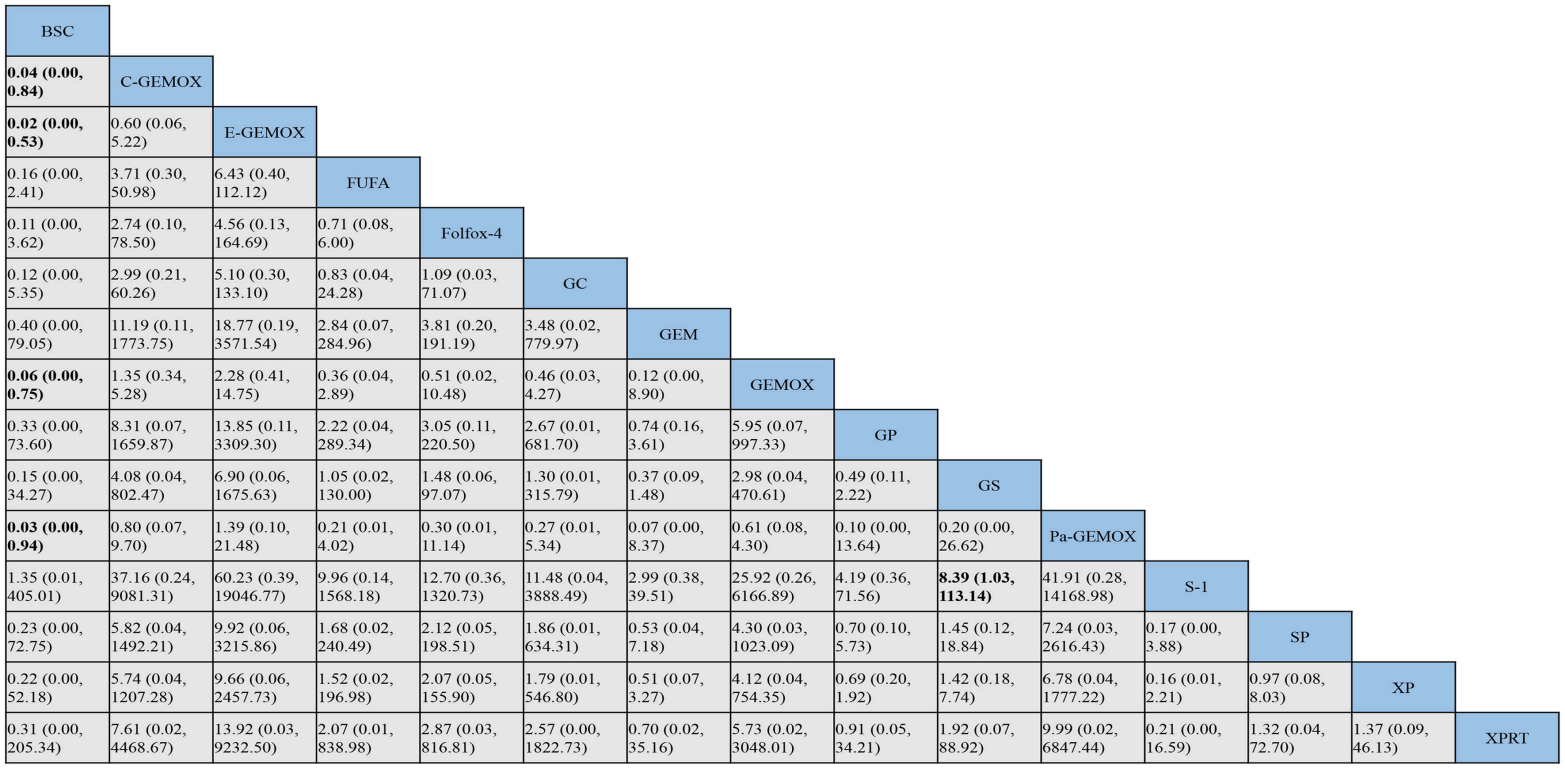
Bayesian framework network meta-analysis. Odds ratio and 95% confidence intervals for objective response rate.

The evidence network of eligible comparisons for OS, PFS and ORR in this NMA is shown in (Figures S1–S3). Calculated by Bayesian NMA, the result of indirect comparisons of OS and PFS was expressed with hazards ratio and credibility interval, and ORR was expressed with odds ratio and credibility (Figures 5–**7**).

HRs for overall survival was presented in Figure 5, statistical significance was found when best support care (BSC) compared to GP, GS, cediranib plus GP (GPCe), panitummumab plus GP (GPPa), Folfox-4 and capecitabine plus cisplatin (XP) (HR 2.2, 95% CI 1.2-3.8; HR 2.0, 95% CI 1.2-3.5; HR 2.5, 95% CI 1.1-5.7; HR 3.1,95% CI 1.2-7.6; HR 3.4, 95% CI 1.7-6.7; HR 2.9, 95% CI 1.4-5.7; respectively). Among the above treatments, the Folfox-4 regimen (HR 3.4, 95% CI 1.7-6.7) seemed to achieve superior survival outcomes. Also, when compared to the standard GP regimen, GPCe, GPPa, Folfox-4 and XP were associated with better OS (HR 0.85, 95% CI 0.47-1.6; HR 0.70,95% CI 0.35-1.4; HR 0.65, 95% CI 0.31-1.4; HR 0.74, 95% CI 0.74, 95% CI 0.51-1.1; respectively), among which Folfox-4 (HR 0.65, 95% CI 0.31-1.4) still tended to obtained greater improvement. Gemcitabine plus carboplatin (GC) was approximately obtained the same OS like GP (HR 1.1, 95% CI 0.71-1.5). The treatments of C-GEMOX, GEMOX, erlotinib plus GEMOX (E-GEMOX), panitummumab plus GEMOX (Pa-GEMOX), FUFA, GC, 5-FU and doxorubicin plus mitomycin C (FAM) and BSC were more likely to lead to worse outcome of OS comparing to other treatments. When BSC compared to the treatments of E-GEMOX and GC, the two treatments did not indicate a tendency of superior efficacy in OS (HR 1.0, 95% CI 0.46-2.2; HR 0.82, 95% CI 0.38-1.7; respectively). When it came to targeted agents, we found that targeted agent plus GP regimen could lead to a superior success in OS than GEMOX. For example, when panitunmmumab was used with GEMOX it showed a superior OS than with GP (GPPa vs. Pa-GEMOX: HR 0.42, 95%CI 0.12-1.5).

For progression free survival (Figure 6), statistical significances were found in the comparisons of FAM to C -GEMOX, GEMOX, Pa-GEMOX, and 5-FU and cisplatin plus radiotherapy (FUPR) (HR 4.1, 95% CI 1.4-12.0; HR 3.6, 95% CI 1.4-9.0; HR 4.6, 95% CI 1.4-15.0; HR 5.6, 95% CI 1.5-20.0; respectively) while the FUPR (HR 5.6, 95% CI 1.5-20.0) was the best. Overall, the regimens of C-GEMOX, GEMOX, Pa-GEMOX, FUPR, GPCe, GPPa, GC and XP were associated with better PFS when compared to other treatments. Unlike the result of overall survival, GEMOX plus targeted agents seemed to receive better outcomes than GP. The treatments of GEM, GEM plus sorafenib (GSo), S-1, FUFA, FAM, BSC, and vandetanib (V) had the tendency to achieve inferior PFS. When BSC compared to other regimens, GSo, FUFA and FAM did not show a superior PFS than BSC (HR 1.0, 95%CI 0.35-2.9; HR 1.0, 95% CI 0.24-4.4; HR 0.74, 95% CI 0.25-7.3; respectively). The regimen of Folfox-4 did not show its advantage in PFS and it was still showing a better efficacy than the standard GP regimen (HR 0.89, 95% CI 0.33-2.4).

As for objective response rate (Figure 7), when compared to BSC, the treatments of C-GEMOX, E-GEMOX, GEMOX, Pa-GEMOX generally obtained a better ORR with statistical significance (OR 0.04, 95% CI 0.00-0.84; OR 0.02, 95% CI 0.00-0.53; OR 0.06, 95% CI 0.00-0.75; OR 0.03, 95% CI 0.00-0.94; respectively) while E-GEMOX (OR 0.03, 95% CI 0.00-0.94) showed a higher incidence of ORR. Though inferior than regimens containing targeted agents, the Folfox-4 regimen is more likely to react than GP (GP vs. Folfox-4: OR 3.05, 95%CI 0.11-220.50). Conversely, S-1 monotherapy was associated with a trend of inferior ORR comparing to BSC (OR 1.35, 95% CI 0.01-405.01). The ORR was likely to be lower in patients treated with Gem, GP, XP plus radiotherapy (XPRT) and slightly higher in FUFA, XP, S-1, S-1 plus cisplatin (SP) and GS.

### Ranking of the Including Regimens

**Figure 8:**
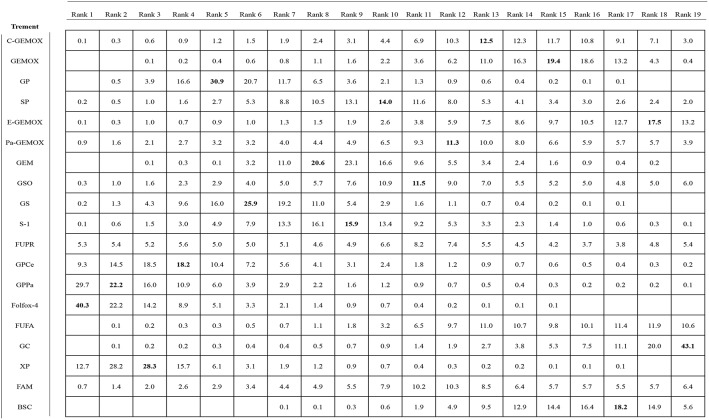
Ranking for overall survival.

**Figure 9:**
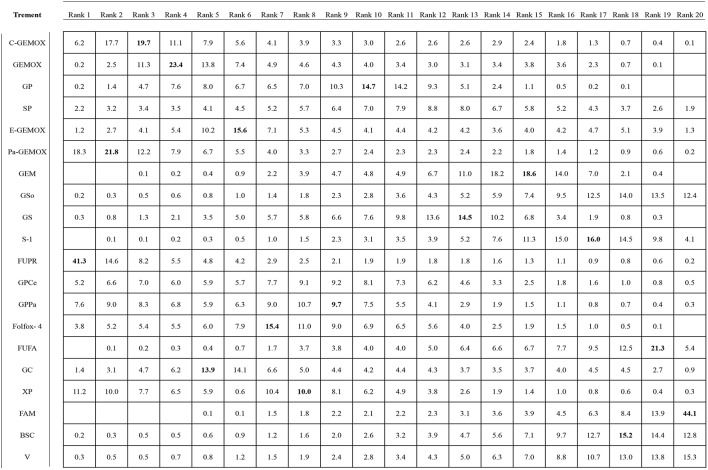
Ranking for progression free survival.

**Figure 10:**
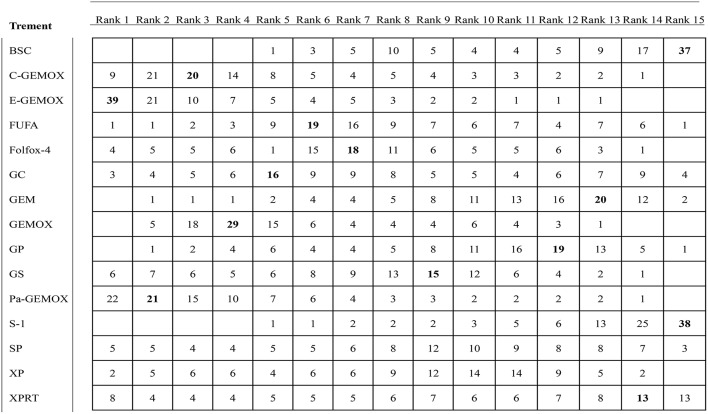
Ranking for objective response rate.

Values of SUCRA were shown in Figures 8–10. As for OS shown in Figure 8, the treatment of Folfox-4 had the largest probability of being the rank 1 (40.3%). GPPa was most likely to be the rank 2 (22.2%), XP to be the rank 3 (28.3%) and GP to be the rank 4 (30.9%) while GC was much more likely to be the rank 19 (43.1%) and E-GEMOX to be rank 18 (17.5%). With respect to PFS, the FUPR had the highest tendency to be the rank 1 (41.3%), Pa-GEMOX to be the rank 2 (21.8%), and C-GEMOX to be the rank 3 (19.7%) and GEMOX to be the rank 4 (23.4%). The treatment that had the largest probability to be the rank 20 was FAM (44.1%) and FUFA was likely to be the rank 19 (21.3%) (Figure 9). The Figure 10 revealed that E-GEMOX was most likely to be the rank 1 (39%) for ORR, Pa-GEMOX to be the rank 2 (21%), C-GEMOX to be the rank 3 (20%) and GEMOX to be the rank 4 (29%). BSC (37%) and S-1(38%) were likely to be rank 15 and the treatments XPRT was likely to be the rank 14 (13%).

## Discussion

In this systematic review and NMA conducted to find the optimal chemotherapy for patients with advanced biliary tract cancer, we made several key observations. Firstly, for targeted therapy in this study, therapies targeting EGFR in this study contained erlotinib, cetuximab and panitumumab. Cediranib and sorafenib targeted VEGFR while vandetanib targeted EGFR/VEGFR. Different targeted therapies seem to lead to different efficacy. Our study has come to the conclusion that the targeted therapy targeting EGFR was superior than targeting VEGFR in PFS which was ever illustrated in two meta-analysis (16, 17). Though targeted therapy is currently unsatisfied in prolonging patients' survival, targeted therapy is still a hot topic. New mutations accounting for biliary tract cancer have been reported and published, stimulating the emergence of new targeted therapies (42). Secondly, Folfox-4 regimen, combined oxaliplatin, 5-FU and folinic acid, appeared to obtain a better OS in our study. The Folfox regimen was initially used in the treatment of colorectal cancer and it was first found to obtain positive efficacy in a prospective phase II study in Korea using the Folfox-6 regimen (43). A recent randomized phase II study of first-line treatment for advanced biliary ducts carcinoma demonstrated that the chemotherapy of Folfox-4 produced a desired result without increasing adverse events when comparing with FUFA (HR for OS, 0.3121, 95% CI, 0.1535-0.6345, *P* = 0.0031) (9). Oxaliplatin, a third-generation platinum analog, is convinced in previous studies (16, 17) that it can achieve improvement in PFS and ORR with less toxicity but failed to prolong OS. But in our study, we found that oxaliplatin combined with 5-FU and folinic achieved superior success in OS and it ranked first in the value of SUCRA. The regimen of Folfox-4 may be a better alternative to the standard GP regimen. Last but not least, most of the including treatments were mainly different combinations made by gemcitabine, platinum (cisplatin, carboplatin and oxaliplatin) and fluoropyrimidine (5-FU, S-1 and capecitabine). In our study, gemcitabine plus platinum regimens were generally found to obtain better efficacy than fluoropyrimidine based regimens. A previous NMA (11) came to the same conclusion that gemcitabine based regimens yielded more satisfying survival outcomes than fluoropyrimidine based. But when it came to toxicity, a gemcitabine based regimen was likely to cause neutropenia than fluoropyrimidine based regimens. Lastly, GS and XP were found to achieve approximately equal efficacy like the standard GP regimen both in OS and PFS. S-1 and capecitabine are oral form of fluorouracil that is convenient to patients. It was only explained in retrospective studies (24, 31) and no meta-analysis has ever can to this conclusion. Therefore, randomized trials are required to convinced this conclusion.

Recently, there are several clinical trials on going. A study conducted by Kobayashi et al. in Japan revealed that GP plus S-1 had a promising survival benefit (44) and a randomized phase III trial comparing this regimen to GP is underway (KHBO1401, UMIN000014371). A phase III trial comparing GEMOX to capecitabine plus oxaliplatin is in progress in South Korea (NCT01470443). A phase II/III trial of FOLFIRINOX (5-FU plus irinotecan plus oxaliplatin, a standard chemotherapeutic regimen for advanced pancreatic cancer) is also on going in France (NCT02591030). New meta-analysis is needed by then.

This NMA does have some limitations. In order to make indirect comparison available, this analysis included not only RCTs but also retrospective studies which increased the heterogeneity in design. Because of the lack of enough evidence, it was an inevitable problem that the reliability of the result in our study had greatly reduced. Also, most of the studies included did not provide detail information, so a stratified or subgroup analysis in our study was not accomplished. There were variations in the definition of advanced biliary tract cancer which included not only locally advanced or metastasis but also recurrence in this systematic review and some of the studies also could not give a specific illustration of the clinical stage. In this analysis, treatment outcomes of the four tumor types could not be extracted, respectively, and proportion of cancers based on the anatomy location was not balanced. Detailed analysis was hard to perform and we draw the above conclusions on the premise of ignoring the heterogeneity produced by different types' characteristics. And in our study, we assumed that patients with biliary tract cancer in different countries react to the same chemotherapeutic regimen similar. Additional, populations, gender ratio, and patients' age of these studies were assumed similar though they differed among the studies enrolled. For example, some studies got a large sample of up to 410 people while some studies got a small sample like 43 people. The ratio of men and women was approximately equal in only a few studies and the proportion of men was larger than women in all. Patient' age in the majority of our studies was about 65 years old, so attention can be taken away from the heterogeneity produced by age. Publication bias and selective reporting biases cannot be excluded which might affect some comparisons. Also, some treatments could not be included due to failing for obtaining full-text or connecting to other treatments. In addition, articles not published in English were not included which might lead to a language bias. Lastly, direct comparisons were scant in some comparisons which might affect the result of the analysis. Therefore, it seemed that the optimal treatment still remained uncertain and more RCTs are required.

In addition, a meta-analysis conducted by Zhuang (16) has discovered that different tumor types react differently to chemotherapy. In that study, it was found that cholangiocarcinoma patients are more sensitive to chemotherapy gallbladder adenocarcinoma or patients with ampulla of Vater cancer. Patients with cholangiocarcinoma (intrahepatic and extrahepatic) constituted the largest percentage in most studies, almost twice than gallbladder patients. The problem has been raised up but no study has made a clear answer. Besides, biliary tract cancer has a characteristic of regional distribution disparity and the influence of different region has been proven irrelevant. New studies are needed to explore this issue and find the answer.

## Conclusions

From this NMA, we can conclude that targeted therapy currently achieved little success in prolonging OS but is still a promising treatment. The Folfox-4 regimen may be a new replacement for the previous GP regimen as it achieves a lot in OS. XP and GS can be considered as convenient alternatives for advanced biliary tract cancer.

## Data Availability

The raw data supporting the conclusions of this manuscript will be made available by the authors, without undue reservation, to any qualified researcher.

## Author Contributions

YL, YZ, YH, MH, DZ, and FL designed the study. YZ and YH screened studies and collected data. YZ and CY did the statistical analyses. YZ, YH, MH, and SW prepared the figures and reviewed the results. YL, YH, and MH interpreted data. YL and YH wrote the manuscript. YL, CY, DZ, and FL revised the manuscript.

### Conflict of Interest Statement

The authors declare that the research was conducted in the absence of any commercial or financial relationships that could be construed as a potential conflict of interest.

## Supplementary Material

Figure S1 Network evidence of the eligible comparison for overall survival. The width of the lines represents the cumulative number of trials for each comparison and the size of every node is proportional to the number of enrolled participants. Lines connect the interventions that have been studied in head-to-head (direct) comparisons in the eligible studies.

Figure S2 Network evidence of the eligible comparison for progression free survival.

Figure S3 Network evidence of the eligible comparison for objective response rate.

## Abbreviations

BTC: biliary tract cancer
RCT: randomized controlled trial
OS: overall survival
PFS: progression free survival
ORR: objective response rate
HR: hazard ratio
OR: odd ratio
VEGFR: vascular endothelial growth factor receptor
EGFR: epidermal growth factor receptor
SUCRA: surface under the cumulative ranking curve
BSC: best support care
GEM: gemcitabine
OX: oxaliplatin
C: cetuximab
E: erlotinib
Pa: panitummumab
Ce: cediranib
V: vandetanib
RT: radiotherapy
GC: gemcitabine + carboplatin
GP: gemcitabine + cisplatin
SP: S-1 + cisplatin
XP: capecitabine + cisplatin
GSo: gemcitabine + sorafenib
GS: gemcitabine + S-1
GV: vandetanib + gemcitabine
Folfox-4: 5-FU + folinic acid + oxaliplatin
FUFA: 5-FU + folinic acid
FUPR: 5-FU + cisplatin + radiotherapy
FAM: 5-FU + doxorubicin + mitomycin C.
