# Supplementary figures and images for: The Efficacy of Different Chemotherapy Regimens for Advanced Biliary Tract Cancer: A Systematic Review and Network Meta-Analysis
Source: Front Oncol. 2019 May 29;9:441. doi: 10.3389/fonc.2019.00441 (PMC6549535; doi:10.3389/fonc.2019.00441)

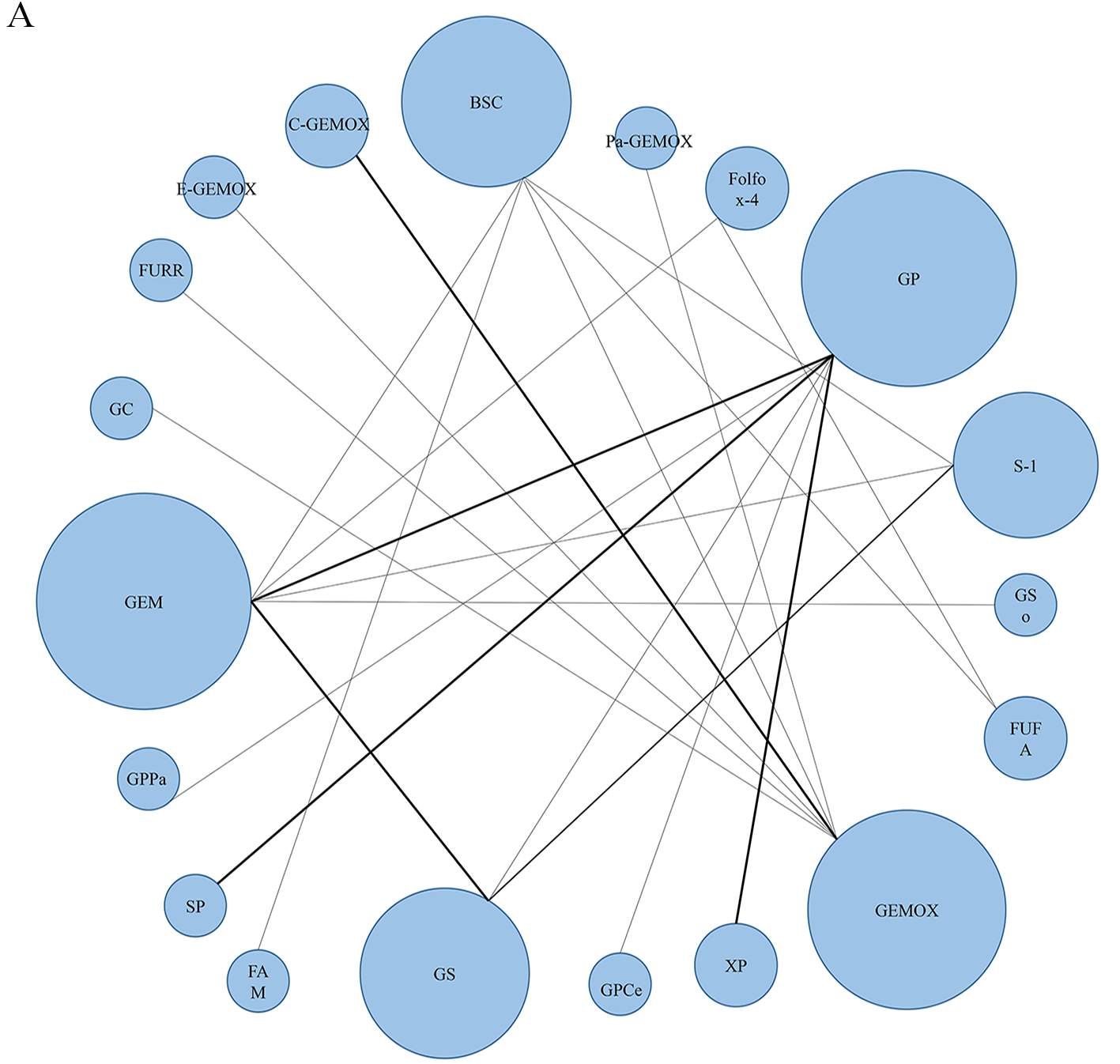

Supplement: Figure S1 — Network evidence of the eligible comparison for overall survival. The width of the lines represents the cumulative number of trials for each comparison and the size of every node is proportional to the number of enrolled participants. Lines connect the interventions that have been studied in head-to-head (direct) comparisons in the eligible studies. [file Image_1.TIF]

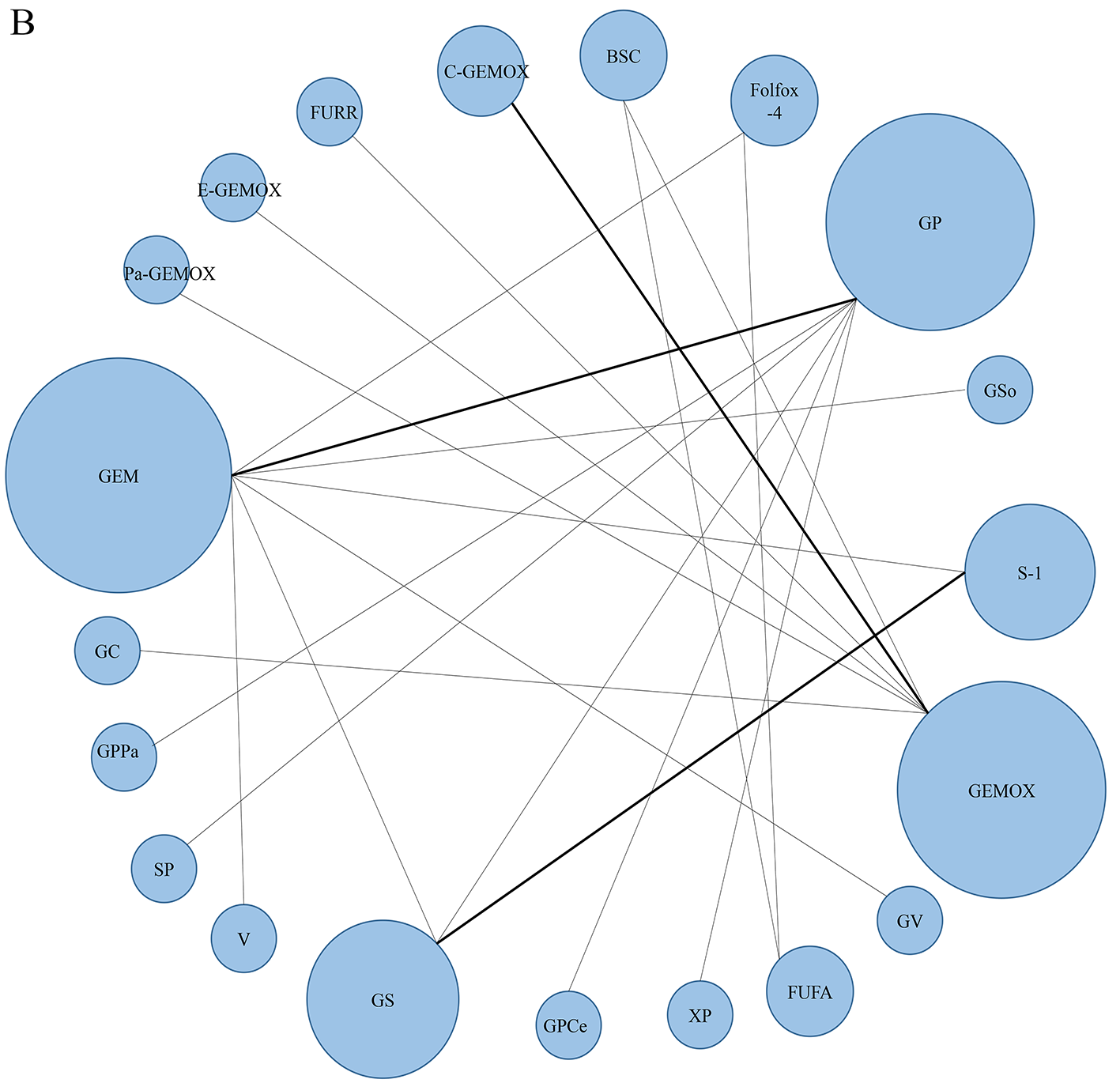

Supplement: Figure S2 — Network evidence of the eligible comparison for progression free survival. [file Image_2.TIF]

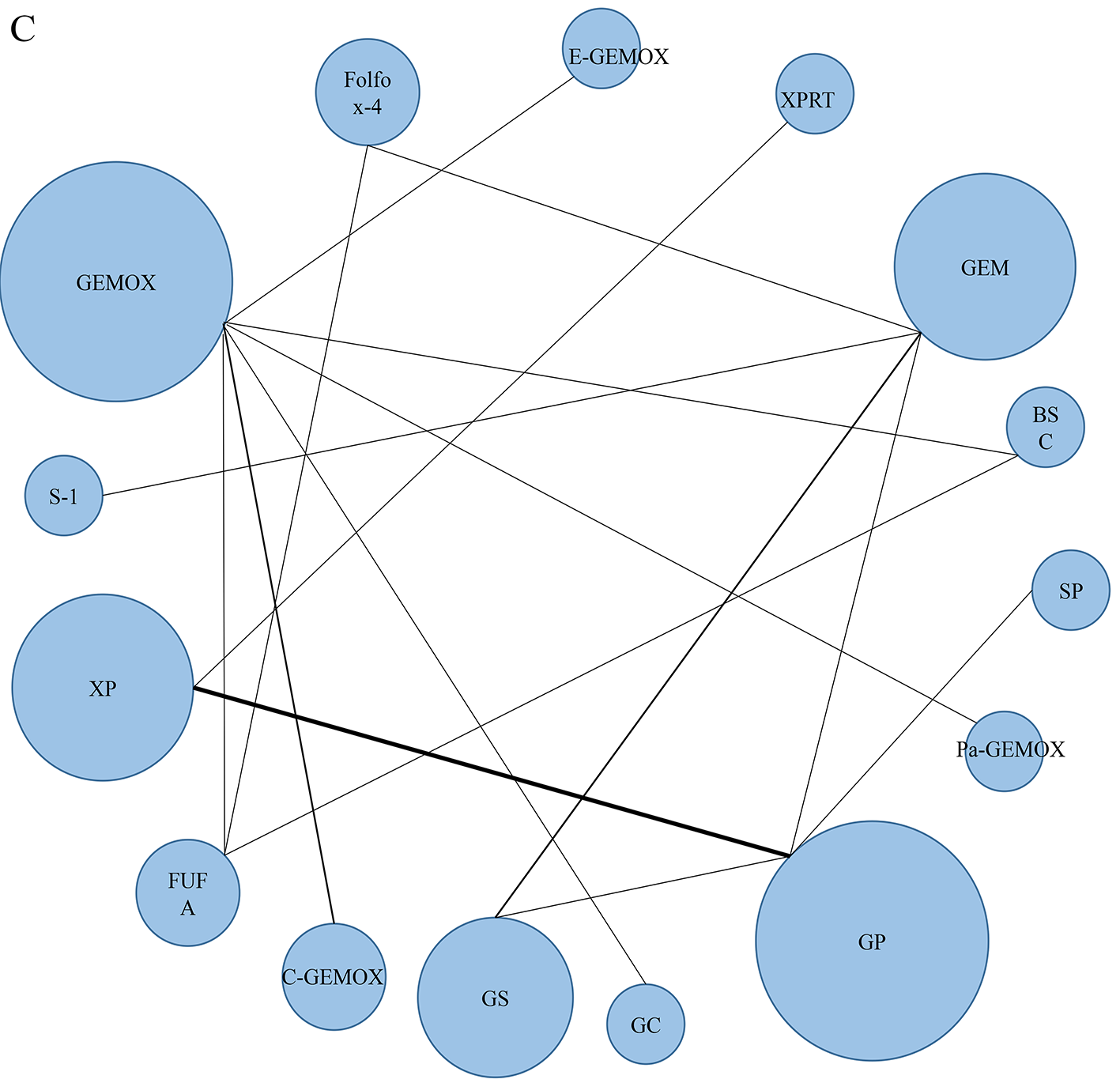

Supplement: Figure S3 — Network evidence of the eligible comparison for objective response rate. [file Image_3.TIF]
